# Common ground: the opportunity of male contraceptives as MPTs

**DOI:** 10.3389/frph.2023.1278709

**Published:** 2023-11-10

**Authors:** Heather L. Vahdat, Logan M. Nickels

**Affiliations:** Male Contraceptive Initiative, Durham, NC, United States

**Keywords:** male contraception, multipurpose prevention technologies, contraception, MPTs, pharmaceutical development, global access

## Abstract

Multipurpose prevention technologies (MPTs) and male contraceptive methods are currently in development to address unique and critical needs facing the global reproductive health community. Currently, MPT products in development are exclusively female-focused due to the readily available nature and regulatory precedent offered by female contraceptive active pharmaceutical ingredients (APIs); however, the opportunity to explore codevelopment with male contraceptive methods, which are at a comparatively early stage of development, should not be overlooked. These fields face parallel challenges including research and development, commercialization, regulatory approval, and market uptake, and these parallels can inform strategic alignment between the fields. One challenge that precludes codevelopment, however, is the path to market and associated funding models for these innovative, yet underappreciated fields. Without candid review, reconsideration, prioritization, and innovation led by the donor and investment communities, product developers will have no compelling reason to consider accepting the added regulatory and fiscal burden associated with combining development streams.

## 1. Introduction

Multipurpose prevention technologies (MPTs) are innovative products in development which combine anti-infective and contraceptive properties, presenting an opportunity to revolutionize public health by simultaneously addressing rates of unintended pregnancies and sexually transmitted infections (STIs). However most, if not all, MPTs in development are meant for use by those that can become pregnant, rather than those that produce sperm. This is a direct reflection of the fact that the majority of marketed contraceptive methods are also designed for women, while novel male contraceptive technologies are still in development. Since most MPTs in development consist of a combination of existing, previously-approved active pharmaceutical ingredients (APIs), MPTs for men are comparatively underrepresented, with no active programs in development.

## 2. Status of male contraception

The development of male contraceptive methods is not a new concept. Scientists have been exploring options for male contraceptives for over 70 years (1). These explorations were overshadowed by the launch of the first female contraceptive method in 1960, when “the pill” and indeed the very concept of contraception, became synonymous with women's reproductive and social autonomy. While these benefits cannot be understated, the responsibility and burden of contraception also became firmly affixed to women. Meanwhile, the only reversible contraceptive method for men continues to be the condom (ironically, the only MPT currently available).

While development of male contraceptives has lagged behind female contraception, the field has seen marked progress in recent years with programs across multiple mechanisms of action advancing to clinical application. A daily-administered hormonal gel and a long-acting injectable hydrogel are currently in clinical trials and several potential products are anticipated to move into clinical trials in the next 12–24 months, including multiple non-hormonal oral pills. These comparatively late-stage programs are in addition to many other projects that are progressing through the earlier stages of development, (i.e., discovery, optimization, preclinical). These early-stage programs are often identifying drug-like compounds and have not yet begun to consider formulations or routes of administration, and therefore are theoretically well-positioned to investigate co-development with other APIs.

Given the evolving nature of the male contraceptive sector, it seems opportune to consider dovetailing MPT products into the existing product development pipeline rather than circling back to create MPT products after the contraceptive products have completed the full development and approval cycle. However, the idea of developing a dual-indication product is daunting both in theory and in practice. The male contraceptive field already faces extremely limited funding and uncharted regulatory pathways. It is understandable that the idea of further challenging the progress of products by attempting to integrate a second indication and API would not be inherently compelling.

## 3. Shared development and funding challenges

The many challenges facing the development of MPTs are well understood, have been recapitulated in multiple publications over the past decade, and are similar to those facing male contraceptives (2–5). Manufacturing and delivery system questions, how to efficiently and effectively design clinical trials, and navigating novel regulatory pathways are all problems faced by MPT and male contraceptive product developers alike. With this overlapping need, combining efforts to engage regulatory agencies to (1) increase awareness that these unique and complementary products are in development and (2) to identify the potential gaps in knowledge that will support the development of better-fitting regulatory guidance is critical. For example, there currently is no male contraceptive-specific guidance from the Food and Drug Administration (FDA). Instead, male contraceptive developers are left to consider existing, tangentially relevant guidelines, patchworking female contraceptive guidance (6) with additional input derived from testicular toxicity guidance (7). Similarly, MPT product developers can reference guidance structured for codevelopment of two or more investigational new drugs (8); however, whether or not this guidance is appropriate depends upon a subjective assessment of the proposed product against defined criteria. Without more tailored guidance it is nearly certain that these products, which address critical global public health needs, will face avoidable, unnecessary, and costly delays on their path to market. Unfortunately, these costs are likely to be passed on to consumers through higher product pricing, which, in turn, limits accessibility.

While the fields of MPT and male contraceptive product development can collaborate to develop strategies to address these challenges, the largest limitation for both is low levels of funding compared to other therapeutic areas. The lack of investment from the pharmaceutical industry, which normally works in concert with public-sector funders (9), has significantly stymied development in both the contraceptive and STI sectors for decades. The resulting gap in funding has partially been filled by the philanthropic sector, specifically foundations and other non-profit organizations; however, their combined fiscal efforts still pale in comparison to industry research and development (R&D) expenditures. For instance, in 2021, funding for MPTs and all contraceptive methods combined was $165 million (Table 1). Pharmaceutical sector investment accounts for 20% of this funding ($23 million) but represents a $16 million decrease from industry investment in 2020. By comparison, the annual budgets for the top 10 pharmaceutical companies in 2021 ranged from $7–$16 billion (11). The reasons for this lack of investment from the pharmaceutical industry likely derive from a perceived lack of market demand and/or from concerns regarding the distinctive regulatory hurdles and legal concerns associated with developing products for a preventative purpose, as is the case with MPTs and male contraceptives.

**Table 1:** Annual funding for MPTs and contraception (2018–2021) (10).

Funding	2018		2019		2020		2021	
	$USD (M)	% Total funding	$USD (M)	% Total funding	$USD (M)	% Total funding	$USD (M)	% Total funding
MPTs	54	0.32	33	0.19	30	0.18	48	0.29
Contraception	114	0.68	143	0.81	140	0.82	117	0.71
Male	9.1	0.08^a^	15.7	0.11^a^	15.4	0.11^a^	12.9	0.11^a^
Female	95.8	0.84^a^	110.1	0.77^a^	107.8	0.77^a^	83.1	0.71^a^
Multiple/Unspecified	9.1	0.08^a^	17.2	0.12^a^	15.4	0.11^a^	19.9	0.17^a^
Total Contraception + MPTs	**$168M**		**$176M**		**$170M**		**$165M**	

^a^ % of total funding for contraception only.

## 4. Discussion: re-evaluating the path to market

As reproductive health is not a priority for most major pharmaceutical companies, the onus lies on the donor and social sector investment communities to lead by example and take action to expand efforts to develop MPTs and the novel contraceptive methods that support them. Sponsoring MPT-specific funding opportunities, convening collaborative workshops to openly discuss regulatory experiences and needs, and advocating for additional funding for public sector grantmakers are all excellent steps, but in order to impart significant change and progress, a major shift in mindset and strategy is needed.

The current pharmaceutical industry model is not a fit for every therapeutic area and serious introspection is required to assess if it is the correct model to be targeting for the contraceptive and MPT sectors. In the current model, based on contraceptive products developed over the past 30 years, the pharmaceutical industry does not invest in R&D until a product has been sufficiently “de-risked”. This model applies in other therapeutic areas as well, but with respect to contraception and MPTs there are a number of incompatibilities that make the traditional pharmaceutical development model unsuitable.

First, there is no definitive milestone at which a product is deemed “de-risked” to the point of being commercially compelling to a pharmaceutical company. These decisions are made behind closed doors at pharmaceutical companies on a case-by-case basis, often weighing profit over impact, which is understandable as profit is the driving force behind the pharmaceutical industry. Second, despite considerable evidence (12, 13), there are still questions regarding whether there are sufficient markets for MPTs and male contraceptives to make pharmaceutical investment worthwhile. Finally, given the fact that the philanthropic and public sectors are essentially carrying these products through a significant level of development, their associated missions and interests become entwined with the products they are supporting. For instance, organizations like Male Contraceptive Initiative support missions for global access and affordability of products developed with their funds. This mission-driven approach can result in conflict with the traditional pharmaceutical sector approach which is driven primarily by profit. While efforts have been made over the past decades to find common ground (e.g., tiered pricing models, market shaping efforts in low- and middle-income countries) there are still significant delays in the time that it takes products to reach vulnerable populations (14).

While the lack of pharmaceutical investment is the most discussed financial barrier for the development of male contraceptives and MPTs, the current R&D model conflicts with the MPT and contraceptive sectors even earlier in the product development process. Venture capital (VC), often the precursor to pharmaceutical investment, is critically lacking. As with the pharmaceutical industry, one major challenge faced in attempting to attract investors is a lack of understanding or underappreciation of the potential markets for male contraceptives and MPTs. In addition, VCs traditionally move quickly with a general expectation of holding an investment in their portfolio for 3–8 years over a timeline from discovery to the end of Phase II clinical (15). It is about this point where the baton is often handed off to a pharmaceutical partner; the VC's willingness to assume early risk rewarded by return on investment derived from the pharmaceutical partner's investment to obtain licensing fees or direct purchase of an asset. As such, if either the VC or pharmaceutical partner (or both as is often the case in the contraceptive and MPT sectors) is not present, the handoff chain, as well as the path to market, breaks down dramatically.

This model presents a particularly conflicting expectation for contraceptive products designed to offer longer-term prevention, known as long-acting reversible contraceptives (LARCs). For any product designed to be a LARC, or in the case of MPTs that are combined with a LARC, there is an inherent impact on the development timeline, particularly during clinical phases. For instance, if a contraceptive is targeted for use over 3–5 years, then, generally speaking, trial participants will need to be followed for that period of time. If you add in recruitment and time and analysis, the traditional 3–8 year turn around for VC investment is quickly surpassed.

For these reasons, it is important to take a more pragmatic view of the situation facing product developers in the MPT and contraceptives sectors. Rather than continuing to spend the limited resources available on what may be a “square peg in a round hole” scenario out of fear of losing the little ground that has been gained, taking a beat to consider novel approaches may result in long-term gains, particularly in a global context.

## 5. Conclusion

Combining contraceptive and anti-infective agents to develop MPT products stands to offer considerable impact as a more efficient means of addressing two of the biggest challenges facing global health: unintended pregnancy and STI transmission. However, significant challenges lie ahead of these products before they can successfully make their way to the market. Two of the most critical challenges to be addressed are clarifying the regulatory pathway and exploring system-level change to move towards an alternative path in lieu of the traditional pharmaceutical industry and investment models. These challenges are particularly aligned for the male contraceptive and MPT sectors. As such, these sectors can and should combine efforts to: (1) engage regulatory agencies to establish product-specific guidelines to ensure time and cost efficiency, (2) highlight and communicate the ways in which the current pharmaceutical model is not meeting the needs of the MPT and contraceptive sectors, (3) identify and propose modified or novel models to address this disconnect, and (4) and to solicit and educate investors on the unique needs and potential impact offered by the contraceptive and MPT sectors.

## Data Availability

Publicly available datasets were analyzed in this study. This data can be found here: https://policy-cures-website-assets.s3.ap-southeast-2.amazonaws.com/wp-content/uploads/2023/03/01082056/Snapshot-Contraception-RD-Broadening-Horizons-to-Meet-a-Diversity-of-Needs.pdf. Policy Cures Research: G-FINDER.
